# Comparison if the addition of multilevel vertebral augmentation to conventional therapy will improve the outcome of patients with multiple myeloma

**DOI:** 10.1186/s13013-016-0107-6

**Published:** 2016-12-29

**Authors:** Ziad A Audat, Mahmoud H. Hajyousef, Mohammad D. Fawareh, Khaldoon M. Alawneh, Mohannad A. Odat, Mohammad M. Barbarawi, Ali A. Alomari, Rami A. Jahmani, Mohammad A. Khatatbeh, Mohammed A. Assmairan

**Affiliations:** 1 Orthopedic Department, Jordan University of Science & Technology, King Abdullah University Hospital, Irbid, 22110 Jordan; 2 King Abdullah University Hospital, Amman-Ramtha Road, Irbid, 22110 Outside of the US Jordan; 3 Ibn Alhaytham Hospital, Tela’ Al Ali, Amman, 11953 Outside of the US Jordan; 4 Jordanian Royal Medical Services, King Abdullah the second, Amman, 11822 Amman Jordan

**Keywords:** Multiple myeloma, Vertebral augmentation, Vertebroplasty, Kyphoplasty, Chemotherapy, Radiotherapy, Spine, Outcomes

## Abstract

**Background:**

This was a prospective study to evaluate the effect of multilevel vertebral augmentation in addition to conventional therapy in multiple myeloma patients.

**Methods:**

We treated 27 patients, whom were recently diagnosed to have multiple myeloma by two ways of treatment. Thirteen patients (group I) were treated with conventional therapy and 14 patients (group II) with adding vertebroplasty and kyphoplasty. Patients were evaluated pre-treatment and at half, one, two and 3-years post-treatment by using Oswestry Disability Index (ODI), the Stanford Score (SS) and the Spinal Instability Neoplastic Score (SINS).

**Results:**

Mean values of ODI, SS and SINS were 31.9 (63.8%), 4.3 and 13.8 for group I and 33.2 (66.4%), 4.6 and 12.8 for group II before starting treatment. Group II showed improvement better than group I at all follow-up intervals with best results at first 6 months. *P*-values at the end of the study were ODI = 0.047, SS = 0.180 and SINS = 0.002. Mortality rates were equal of both groups (four patients of each group).

**Conclusion:**

Adding vertebral augmentation to conventional therapy improves multiple myeloma patients’ quality of life, but didn’t affect the mortality rate.

## Background

Multiple myeloma is accumulation of malignant plasma cell in the bone marrow leading to impaired blood cell formation and multiple lytic lesions in the skeleton. The incidence of bone involvement is about 70–100% while vertebral column is about 60% [1–3]. Bone becomes week and easy to fracture, which may cause pain in the bone and inability to use the limb. In the spine, fractured vertebra causes pain, kyphotic or kyphoscoliotic deformity, compression of the spinal cord or cauda equina in addition to the general symptoms of multiple myeloma [1, 3].

General treatment of the disease includes radiotherapy, chemotherapy and bisphosphonate to decrease bone resorption in addition to analgesia, bed rest and bracing to treat pathological fractures [1]. Minimally-invasive vertebroplasty and balloon kyphoplasty are used as local treatment of the vertebral lesions to decrease pain and prevent or treat deformities [3–5]. Vertebroplasty is insertion of bone cement (polymethylmetacrylate) inside the vertebral body using pedicle cannula unilaterally or bilaterally while balloon kyphoplasty is insertion of balloon tamps through pedicle cannulae to reduce the height of the vertebra, realign the sagittal plane and create a cavity for bone cement [6–8]. Many studies were concentrating on multilevel vertebroplasty and kyphoplasty to treat multiple myeloma, but not more than 6 and 8 levels [9, 10].

At our hospital, we used vertebral augmentation in the management of multiple myeloma in a different way. We perform multilevel vertebral augmentation for all vulnerable vertebrae; thoracic, lumbar and sometimes the first sacral vertebra. Our practice is to not wait until the vertebra collapsed as a cause of the tumor, which may lead to neurological sequelae. As such, the following prospective study evaluated the outcomes of our multiple myeloma patients who underwent multilevel vertebral augmentation in addition to conventional therapy.

## Methods

This is a prospective study of effectiveness of the addition of vertebral augmentation to conventional chemotherapy and radiotherapy in treating multiple myeloma patients. Our main aims were to prevent spinal column collapse, back deformity, neurological deficits, minimize pain and decrease general morbidities.

**Fig. 1:**
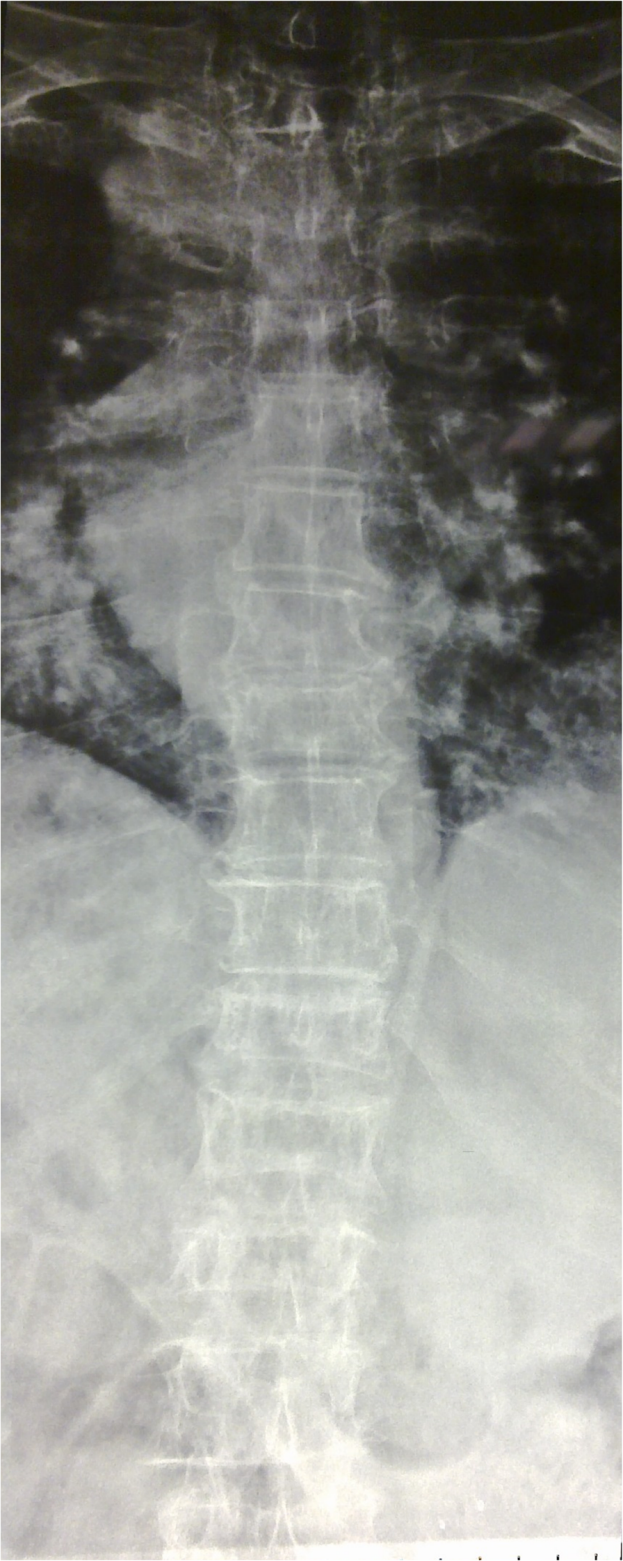
Anteroposterior view of thoracic and lumbar spine

**Fig. 2:**
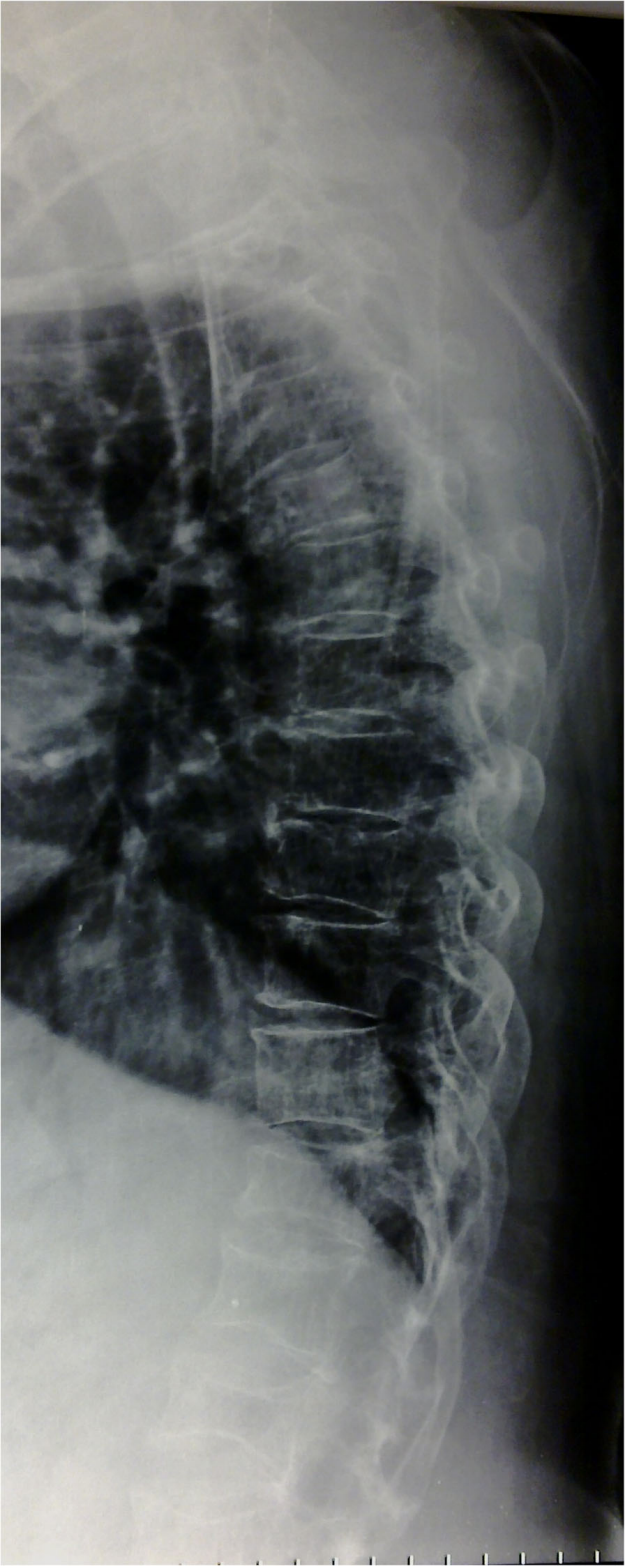
Lateral view of thoracic spine shows vertebrae compressed fractures and some lytic lesions

**Fig. 3:**
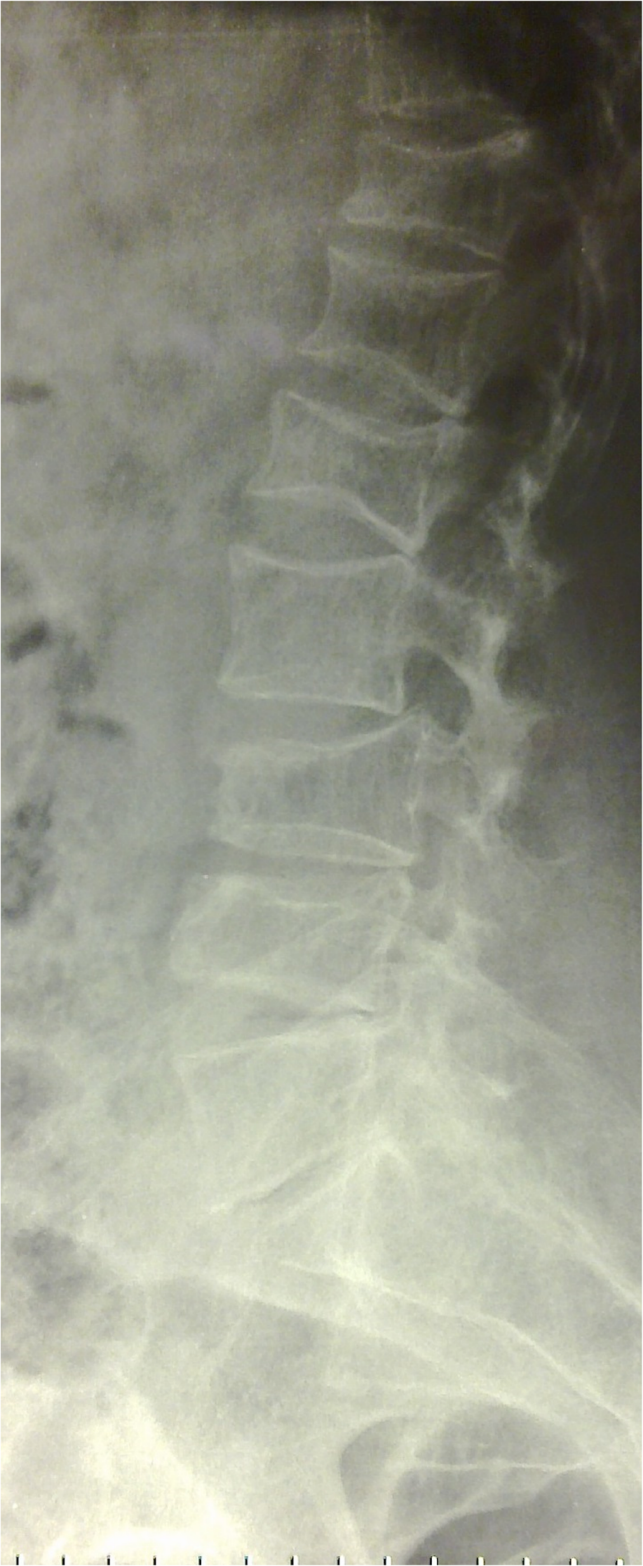
Lateral view of lumbar spine shows compressed fractures and lytic lesions

**Fig. 4:**
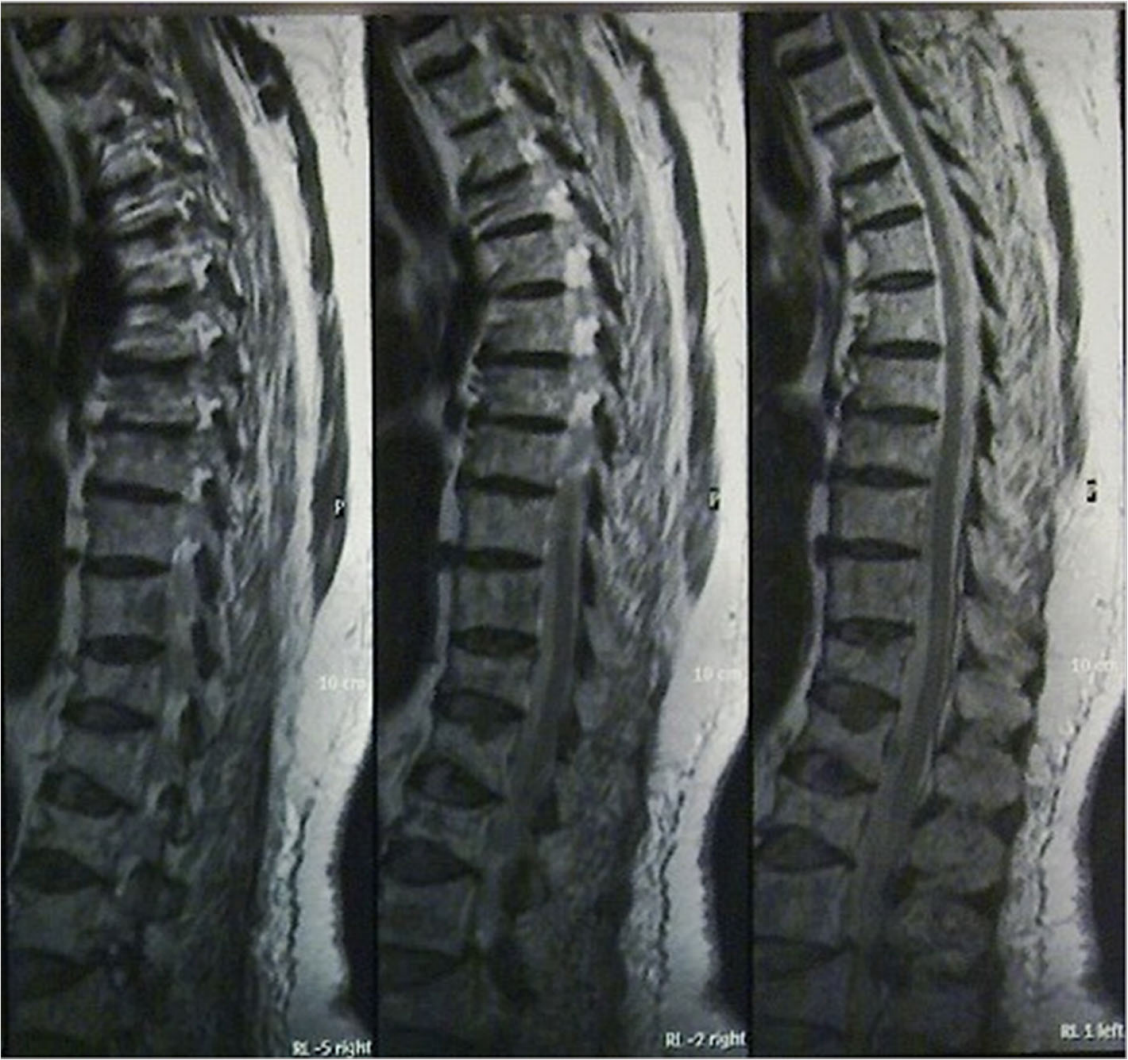
Sagittal views of thoracic and lumbar spine showed multiple lesions of the spine and fracture of many vertebrae

We treated 27 patients diagnosed with multiple myeloma at our institution that were newly diagnosed with more than 3 years follow-up. All patients had back pain without neurological deficits. All patients’ demographic data were extracted from the medical charts, consisting of age, gender, presenting symptoms, and follow-up period. Imaging studies included plain x-ray and magnetic resonance image (MRI) at the time of diagnosis (Figs. 1, 2, 3 and 4). The patients had histological diagnosis with bone marrow biopsy. The involved vertebra included lesions in the thoracic, lumbar, sacral vertebrae and cervical in one patent (C6 and C7). Mild kyphosis was seen in half of the patients. Consent form was signed by patients and Institutional Review Board (IRB) approval was obtained.

All patients received conventional chemotherapy and radiotherapy according to standard protocols of the hematology oncology. Patients were then were randomly categorized into two groups:

Group I: 13 patients were treated by conventional treatment (i.e. chemotherapy and radiotherapy) (Table 1).
Group II: 14 patients; 206 vertebrae, number of vertebrae ranged between 10 and 16 (mean: 14.7); were treated by vertebral augmentation in addition to conventional therapy (five patients with chemotherapy and radiotherapy and nine patients with chemotherapy) (Table 1). One patient needed radiotherapy post augmentation.

**Table 1:** Demographic and clinical characteristics of patients according to method of treatment

	Group 1	Group 2
Number of patients	13	14
Age (years)	39–78 (Mean: 58.15, SD: 12.3)	34–74 (Mean: 58.86, SD: 11.99)
Sex (Male : Female)	9:4	6:8
Back Pain	All patients	All patients
Neurological Deficit	None	None
Treatment	Chemo and Radiotherapy = 7 Chemotherapy = 6	Vertebroplasty and Kyphoplasty Two hundred six vertebrae (one patient 10 v, one patient 12 v, three patients 14 v, two patients 15, and seven patients 16 vertebrae) in addition to Chemotherapy or Chemo and Radiotherapy

**Fig. 5:**
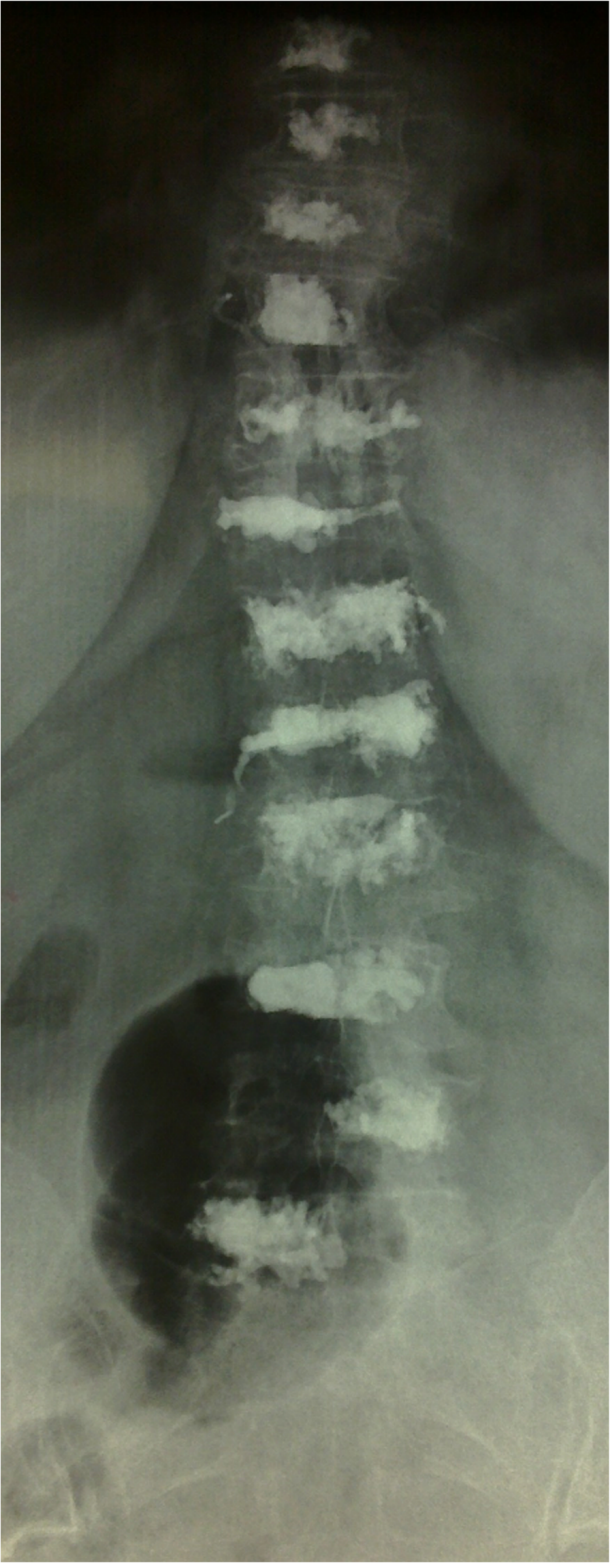
Anteroposterior view of thoracic and lumbar spine with multilevel vertebrae filled with bone cement and lateral small vascular leak

**Fig. 6:**
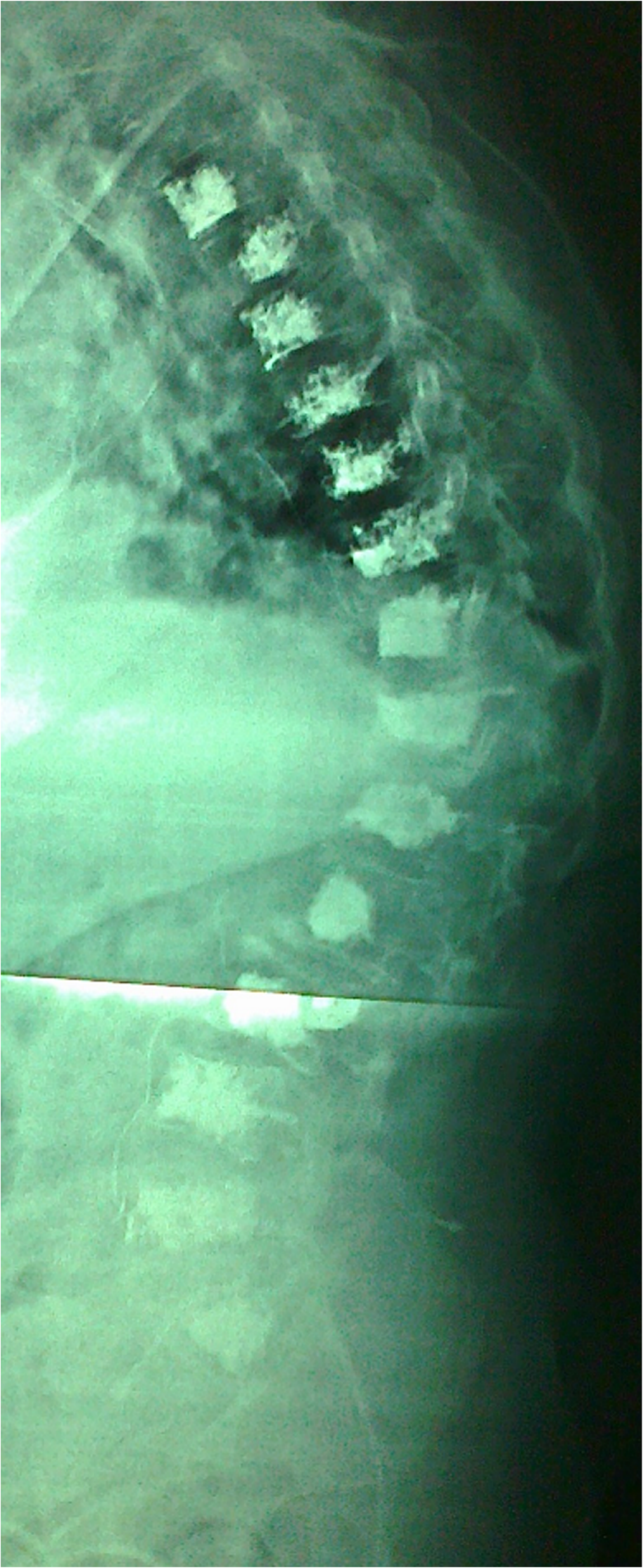
Lateral view of whole spine showed multilevel vertebrae was filled with bone cement and intraspinal cement leak

Vertebral bodies were augmented from the third thoracic (T3) to first sacral vertebrae (S1), all vertebrae were augmented if they were fractured or vulnerable to fracture whatever the size of the lesion. Vertebral augmentation was done under general anesthesia in the operation room under fluoroscope control. All levels for single patient were done at the same session. Balloon kyphoplasty and cement injection was used to restore the height of collapsed vertebra. Two balloons were used for each level. Transpedicular technique was used for vertebrae below T8 and extrapedicular for T8 and above. Vertebroplasty was used for non-collapsed vertebrae by inserting working cannula and injection of bone cement. We used a transpedicular technique for vertebrae below T8 and extrapedicular for T8 and above, unilateral working cannula for T9 and above and bilateral for T10 and below (Figs. 5 and 6). Patients were observed at surgical flour for one postoperative day then transferred to the hematology ward or discharged, then followed-up at the outpatient clinic.

Patients who had spinal canal extension or spinal cord compromise, cauda equine compression, late-stage disease and patients who are previously underwent spinal surgery were excluded. International scoring and questionnaire systems consisting of the Oswestry Disability Index (ODI), the Stanford Score (SS) and the Spinal Instability Neoplastic Score (SINS) were used to evaluate the clinical and radiological results. The patients were evaluated clinically and radiographically on discharge-day, 6, 12, 24 and 36 months.

Statistically, we used SPSS version 20 (Chicago, IL, USA) to evaluate the results. Levene’s test for equality of variances was used to evaluate patients at each follow-up interval. This test gives mean values, standard deviation and *p*-value. Test of between-subjects effect, transformed variables: Average using ANOVA Method to evaluate the end results.

## Results

We treated 27 patients, whom were recently diagnosed with multiple myeloma. There were 13 patients in group I (conventional treatment) and 14 patients in group II (*n* = 206 vertebrae, vertebral augmentation group). Mean age for group I was 58.2 years, mean follow-up was 36 months and male to female ratio was 9:4. For group II, the mean age was 58.9 years, the mean follow-up was 36 months and male to female ratio was 6:8. There was no significant statistical difference in the age between two groups as shown in Table 1.

Four patients (30.8%) of group I died between 7 and 11 months after diagnosis: three patients due to advanced disease and one by acute pneumonia. Four patients (28.6%) of group II died: one died at day of surgery by acute lung embolism and three died 18–24 months after surgery due to advanced disease and severe pneumonia (Table 2).

**Table 2:** Morbidity and mortality of each group

	Group 1	Group 2
Morbidity	Threepatients bed ridden due to multiple spinal fracture and paraparesis. Four patients can walk with aid	All still mobile without aid except one who needs stalk aid.
Mortality	Five patients: four deaths due to advancement of the disease and one due to sepsis after 7–11 months of treatment (38.5%)	Four patients: one at the same day of surgery due to PE, two after 1.5 year of treatment due to advancement of the disease and one after 1.5 years due to pneumonia. (27.3%)
Back Pain Improvement	Six patients improved partially	All patients improved after Vertebroplasty with three of them had episodes of pain.
Vertebroplasty Intraoperative Complications	----	One had small leak toward the spinal canal without neurological disorders. One had intravascular leak.

There were few intraoperative complications in group II. Cement leak inside the spinal canal with no significant neurological compromise or deficits occurred in one patient and intravascular leak in a small vessel was seen in two patients (Figs. 5 and 6). Bone cement didn’t affect chemotherapy or radiotherapy.

Oswestry Disability Index, Stanford score and the Spinal Instability Neoplastic Score values were nearly equal in both groups before treatment. ODI of group I was 31.9 (63.8%) with SD = 8.34 and of group II was 33.2 (66.4%) with SD = 5.98 (*p* = 0.418). SS of group I was 4.3 (SD = 2.6) and of group II was 4.6 (SD = 2.9) (*p* = 0.309). SINS of group I was 13.8 (SD = 2.9) and of group II was 12.8 (SD = 2.9) (*p* = 0.482).

At 6 months-follow up, group II value improved, ODI mean values were 32.5 (64.9% and SD = 10.92) and 23.14 (46.28% and SD = 8.49) for group I and II, respectively (*p* = 0.316). The SS value was 5.26 (SD 2.96) for group I and 7.18 (SD 1.39) for group II (*p* = 0.05). SINS was 12.85 (SD 2.79) for group I and 7.15 SD 3.24 for group II (*p* = 0.449).

At 1 year follow-up, group I score values showed more improvement. ODI value for group I was 28.4 (56.8%) with SD = 8.79 and for group II was 21.4 (42.8%) with SD = 9.24 (*p* = 0.874). The SS value for group I was 5.28 with SD 2.88 and for group II was 7.52 with SD 1.48 (*p* = 0.012). SINS value for group I was 12.85 with SD 2.88 and for group II was 7.23 with SD 3.37 (*p* = 0.526).

At 2 years follow-up, ODI for group I was 28.42 (56.85%) with SD 8.79 and for group II was 21.43 (42.65%) with SD 9.24 (*p* = 0.874). The SS for group I was 5.40 with SD 2.83 and for group II was 7.68 with SD 1.56 (*p* = 0.047). SINS value for group I was 12.75 with SD 2.67 and for group II was 7.31 with SD 3.43 (*p* = 0.278).

At 3 years follow-up, ODI for group I was 29.17 (58.34%) with SD = 9.37 and for group II was 21.43 (42.86%) with SD 9.931 (*p* = 0.840). The SS mean value for group I was 5.27 with SD 2.94 and for group II was 7.83 with SD 1.64 (*p* = 0.040). SINS mean value for group I was 12.58 with SD 2.75 and for group II was 7.36 with SD = 3.72 a (*p* = 0.121).

At the end of the study (3 years), we used test of between-subjects effect, transformed variables: average using ANOVA method to compare the end results of each group. ODI and SINS showed significant difference between two groups (*p* = 0.047 and *p* = 0.002) with less significant difference by using SS (*p* = 0.180). All group II were freely mobile except one who used a cane when walk. All patients were back pain free except three, who had number of exacerbations of pain that may be attributed to disc disease or fracture of vertebral end plate over bone cement. All patients had preserved vertebral height and sagittal balance except for one who had history of inter-scapular pain 4 years after surgery and x-rays showed mild loss of height of T4 around the bone cement which was insignificant as compared to three patients of group I who became bedridden due to vertebral fractures with involvement of the spinal canal.

## Discussion

**Fig. 7:**
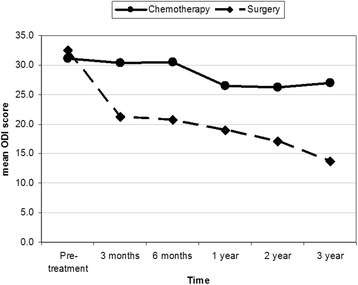
Shows Oswestry Disability Index; pre-treatment and at intervals of follow up

**Fig. 8:**
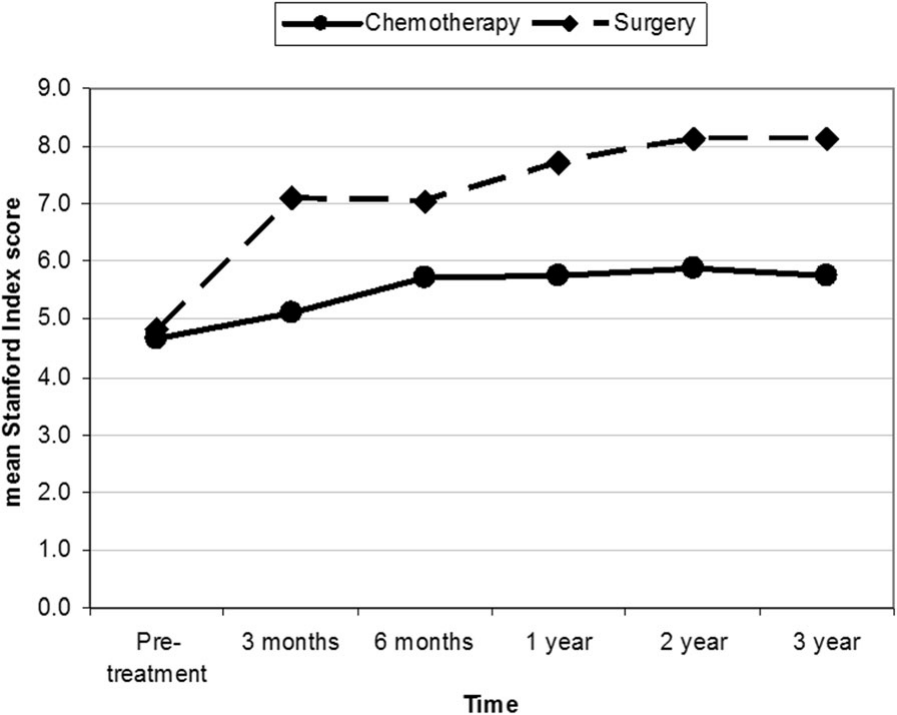
Shows the Stanford Score; pre-treatment and at intervals of follow up

**Fig. 9:**
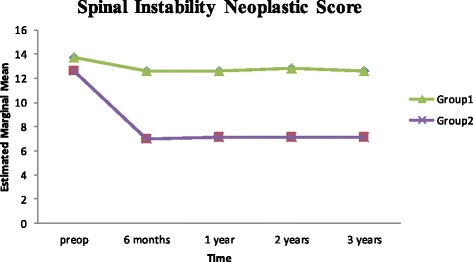
Shows the Spinal Instability Neoplastic Score; pre-treatment and at intervals of follow up

This is was a prospective study to compare two groups of multiple myeloma patients who were treated at our institute. Group I was treated with conventional therapy and group II with multilevel vertebral augmentation in addition to conventional therapy. We couldn’t find any similar studies in the literature doing the same comparison. Most of previous studies involving multiple myeloma discussed mixed population of malignancy and were not focused such a disease. Few studies that focused on multiple myeloma treated only the fractured vertebra and showed similar results of our study [9, 11–17]. Our results showed that the addition of vertebral augmentation had better improvement in the outcome, both subjective and objective. The best rates of scores improvement were seen during the first 6 months. After that, improvement rate decreased with time, whereby group II showed better results as demonstrated in Figs. 7, 8 and 9.

The *p*-values (Levene’s test) of scores at each interval of follow-up is shown in Table 3. *P*-value of ODI was 0.316 at 6 moths and increase to 0.87 at all interval-follow up, which was considered insignificant. *P*-values of SS were ≤0.05 at all follow up periods, which were considered significant. *P*-value of SINS was 0.45 and decreased after 2 years to reach 0.12, which is more significant than ODI. At the end of study, *p*-value (by using ANOVA test) of ODI, SS scores, and SINS were 0.047, 0.180 and 0.002, respectively, which were statistically significant. This means that back pain, mobility, kyphotic deformity due to vertebral collapse and sagittal balance were improved. Most patients of group II became ambulating and totally pain free. As compared to none of group I were pain free and half of them were ambulating with aid.

**Table 3:** Postoperative intervals in relation to outcome scores

	Postoperative Interval			
Outcome Score	6 months	1 year	2 years	3 years
ODI	0.316	0.874	0.874	0.87
SS	0.050	0.012	0.047	0.04
SINS	0.449	0.526	0.278	0.121

Note, T-tailed p-value of each follow-up interval and test of between-subjects effect

*ODI* Oswestry Disability Index, *SS* Stanford Score, *SINS* Spinal Instability Neoplastic Score

In the literature review addressing previous studies discussing multilevel vertebral augmentation, we found that most of them were dealing with less than eight levels and were performed in more than one surgical session [9, 18]. Two case reports were found with multilevel vertebral augmentation. The first one was used to treat newly adjacent level fractures in a patient who was treated for osteoporotic fracture [19]. The second case was treated for multiple osteoporotic fractures that occurred at different times after vertebroplasty in a patient with chronic liver disease [20].

In our study, all the patients had the same disease, were treated by the same hematologist, all procedures were done by the same spine surgeon and evaluated by independent physicians. In group II, 14 patients (206 vertebrae), who underwent vertebral augmentation, the procedures were done at same session for all involved vertebra for single patient. This decreased the need and risk of repetitive anesthesia, although increased the operative time and radiological exposure. We did several measures to decrease surgical time and radiological exposure, inserting a working cannula directly (eliminating introducing cannula and K wire need), inserting multiple cannulas at the same time and using unilateral cannula at T9 and above. In addition, there was no significant statistical difference of mortality rate between the two groups.

## Conclusion

Multilevel vertebral augmentation in addition to conventional therapy showed superior results as compared with conventional therapy alone. It relieves pain, preserves vertebral height, sagittal balance and improves mobility of the patients. There was no significant difference of mortality rates between the two groups, but there was significant improvement of morbidity rates. The limitation of this study was the small sample size and variable follow-up periods. Larger, prospective studies are needed to further assess the outcome of such treatment modalities in multiple myeloma patients.
